# High-pressure thermal conductivity and compressional velocity of NaCl in B1 and B2 phase

**DOI:** 10.1038/s41598-021-00736-2

**Published:** 2021-10-29

**Authors:** Wen-Pin Hsieh

**Affiliations:** 1 Institute of Earth Sciences, Academia Sinica, Nankang, Taipei, 11529 Taiwan; 2 Department of Geosciences, National Taiwan University, Taipei, 10617 Taiwan

**Keywords:** Mineralogy, Condensed-matter physics

## Abstract

Sodium chloride (NaCl) is an important, commonly used pressure medium and pressure calibrant in diamond-anvil cell (DAC) experiments. Its thermal conductivity at high pressure–temperature (*P–T*) conditions is a critical parameter to model heat conduction and temperature distribution within an NaCl-loaded DAC. Here we couple ultrafast optical pump-probe methods with the DAC to study thermal conductivity and compressional velocity of NaCl in B1 and B2 phase to 66 GPa at room temperature. Using an externally-heated DAC, we further show that thermal conductivity of NaCl-B1 phase follows a typical *T*^−1^ dependence. The high *P–T* thermal conductivity of NaCl enables us to confirm the validity of Leibfried-Schlömann equation, a commonly used model for the *P–T* dependence of thermal conductivity, over a large compression range (~ 35% volume compression in NaCl-B1 phase, followed by ~ 20% compression in the polymorphic B2 phase). The compressional velocities of NaCl-B1 and B2 phase both scale approximately linearly with density, indicating the applicability of Birch’s law to NaCl within the density range we study. Our findings offer critical insights into the dominant physical mechanism of phonon transport in NaCl, as well as important data that significantly enhance the accuracy of modeling the spatiotemporal evolution of temperature within an NaCl-loaded DAC.

## Introduction

Understanding how the extreme environments, such as pressure (*P*) and temperature (*T*), influence the thermal conductivity of a material is of fundamental importance for condensed matter physics^1–4^ and geosciences^5–9^. Precise measurements of materials’ thermal conductivity under extreme *P–T* conditions, however, have long been challenging, primarily due to the difficulties in the experimental techniques, such as an accurate characterization of the temperature profile within a small high-pressure chamber. Recent advances in the combination of time-resolved optical techniques with a diamond-anvil cell (DAC)^8,10,11^ have enabled better determination of materials’ thermal conductivity under extreme conditions. In these measurements, the thermal conductivity of a sample of interest within the DAC is typically derived by comparing experimental data related to the heat diffusion rate through the sample with numerical calculations by a thermal model where the thermal conductivity of the pressure transmitting medium is one of the key parameters. Practically, in high *P–T* DAC experiments, NaCl is a commonly used pressure medium and thermal insulation layer; therefore, knowledge of the thermal conductivity of NaCl at high *P–T* conditions is critically needed in order to accurately model the heat transfer and temperature distribution within the DAC.

NaCl is a simple, prototypical ionic crystal. It is in a face-centered cubic structure (six-coordinated, B1 phase) at ambient conditions and transforms to a body-centered cubic structure (eight-coordinated, CsCl-B2 phase) upon compression to ~ 27–30 GPa^12,13^ at room temperature. In addition to the useful applications to high-pressure experiments, the evolution of NaCl’s thermal conductivity and sound velocity with extreme conditions serves as excellent platforms to test the validity of a theory for thermal transport and lattice dynamics, respectively, shedding lights to their fundamental physical mechanisms. For instance, Leibfried-Schlömann (LS) equation is a simple physical model commonly used to predict the *P–T* dependence of the thermal conductivity of a material. The LS equation was formulated for pure, isotropic dielectric crystals where the anharmonic three phonon scattering between acoustic modes was assumed to be the predominant scattering mechanism for thermal transport^14,15^. Prior studies showed that the LS equation reasonably describes the thermal conductivity of H_2_O ice VII^3^ and MgO^16^ within a volume compression range of ~ 33% and ~ 25%, respectively, at room temperature. Testing the validity of LS equation on a crystal over a wider range of volume compression and even in a high-pressure polymorphic phase is of significant importance for materials physics as well as the studies of Earth’s deep interior, since the high *P–T* conditions in deep Earth could substantially alter thermo-elastic properties of minerals and drive them to different crystal structures. If proved valid across a wide range of volume compression and in high-pressure polymorphs, based on the thermal conductivity and equation of state (EoS) of a mineral at a reference *P–T* condition, one could predict its thermal conductivity at a variety of crystal structures and *P–T* conditions, with critical implications for the heat flux and thermal states in deep Earth^16^.

A number of physical properties of NaCl have been extensively investigated, including phase diagram and phase transition^13,17–19^, EoS^20–24^, and sound velocity and elastic constants^25–29^, etc. Studies on NaCl’s thermal conductivity, however, have been limited to relatively low pressures^30,31^. Though recently McGuire et al*.*^32^ reported relative changes in NaCl’s thermal conductivity as a function of relative density across the B1–B2 transition, a precise determination of the thermal conductivity values at high *P–T* conditions remains unavailable. Lack of such critical data may lead to inaccurate modeling of the temperature evolution in laser-heated or externally-heated DAC experiments, which in turn could give rise to inaccurate thermal conductivity of the sample of interest. In this work, we have precisely determined the high-pressure, room-temperature thermal conductivity and compressional velocity of polycrystalline NaCl to 66 GPa. By performing additional simultaneous high *P–T* thermal conductivity measurements, we further show that the thermal conductivity of NaCl at high pressures scales with the *T*^−1^ dependence, typical of a pure crystal. Pressure evolution of the thermal conductivity and compressional velocity of NaCl over a broad density range allows us to confirm the validity of the LS equation and Birch’s law (a linear relationship between compressional velocity and density), respectively, within the *P–T* range we studied. In addition to identifying that the anharmonic three phonon scattering plays a key role in heat transfer in NaCl crystal, our results here also significantly improve the modeling of heat transfer and thus enhance the accuracy of thermal conductivity measurements under high *P–T* conditions.

## Results and discussions

**Figure 1:**
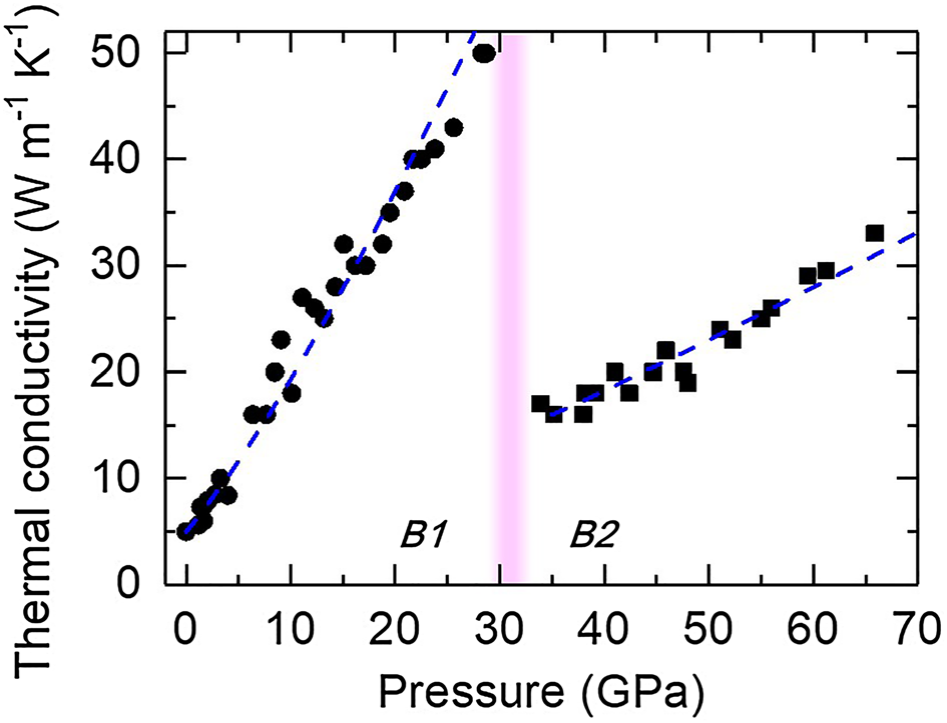
Pressure dependence of the thermal conductivity of NaCl at room temperature. In B1 phase, the thermal conductivity increases rapidly with pressure, while drops upon the B1–B2 transition around 30 GPa (pink shaded region). The increasing rate of thermal conductivity with pressure in the B2 phase is smaller than that in the B1 phase. The experimental uncertainties are typically ~ 20% before 30 GPa and ~ 20–30% at 30–66 GPa. Blue dashed curves represent the predicted thermal conductivity by the LS equation which describes well the data for both B1 and B2 phase (see text for details).

Figure 1 shows the pressure dependence of the thermal conductivity Λ(*P*) of NaCl at room temperature. At ambient pressure, Λ = 5 W m^−1^ K^−1^, which agrees well with literature results^30,31^. The Λ of B1 phase increases rapidly with pressure to 50 W m^−1^ K^−1^ at 28.4 GPa. Upon the phase transition to the B2 phase at ~ 30 GPa, Λ significantly drops to 16 W m^−1^ K^−1^ at 35 GPa, presumably due to the enhanced phonon scattering rate in the eight-coordinated structure with higher anharmonicity^33^. The Λ of B2 phase then increases to 33 W m^−1^ K^−1^ at 66 GPa with a smaller pressure slope (dΛ/d*P*) than the B1 phase. The reduction of thermal conductivity across the B1–B2 transition, almost 70%, is in reasonable agreement with those modeled using several simple interatomic potentials^33^, but much larger than that reported in Ref.^32^ (only ~ 37%), where the reduction was not sampled right across the phase transition.

**Figure 2:**
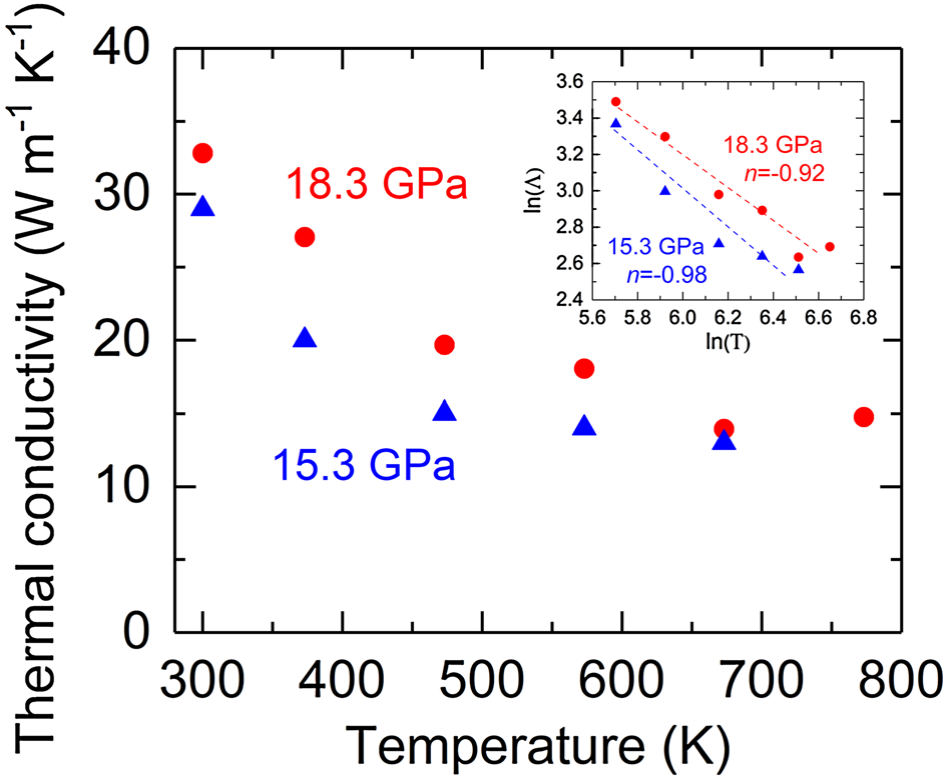
Temperature dependence of the thermal conductivity of NaCl at high pressures. The inset shows the lnΛ-ln*T* plot, where linear interpolation of each set of data derives the exponent of the power law $\Lambda (T) = \alpha \Lambda_{0} T^{n}$, where *n* = −0.98 and −0.92 for the thermal conductivity at 15.3 and 18.3 GPa, respectively. The experimental uncertainties are typically ~ 15–20%.

We have also performed simultaneous high *P–T* thermal conductivity measurements on the NaCl (Fig. 2). Both sets of measurements (*P* = 15.3 and 18.3 GPa) show that the Λ decreases with increasing temperature to ~ 773 K. Note that the heating-induced pressure variations (thermal pressure) were in situ determined to be less than ~ 2–3 GPa, and their effects have been taken into account in the data analysis (Methods). If we assume the thermal conductivity of NaCl follows a temperature dependence $\Lambda (T) = \alpha \Lambda_{RT} T^{n}$, where *α* is a constant and $\Lambda_{RT}$ the thermal conductivity at room temperature, the value *n* is then determined by a linear regression slope in the lnΛ-ln*T* plot (inset of Fig. 2). The resulting *n* is −0.98(± 0.16) and −0.92(± 0.1) for the thermal conductivity at 15.3 and 18.3 GPa, respectively. Both values are close to the typically predicted value of −1 for a pure crystal, indicating that at high pressures, the thermal energy transport in NaCl B1 phase is predominantly controlled by the anharmonic three phonon scattering mechanism^14^.

The Leibfried-Schlömann (LS) equation is often used to model the *P–T* dependence of Λ:

1 $$ \Lambda = A\frac{{V^{1/3} \omega_{D}^{3} }}{{\gamma^{2} T}}, $$

where *V* is the volume,$\omega_{D}$ the Debye frequency, $\gamma$ the Grüneisen parameter, *T* the temperature, and *A* a constant^14,15^. The LS equation has been found to reasonably describe the pressure dependence of thermal conductivity of H_2_O ice VII^3^ and MgO^16^ at room temperature and to ~ 22 and 60 GPa, respectively (corresponding to a volume compression of ~ 33% and ~ 25%). To assess the validity of LS equation on the thermal conductivity of NaCl in B1 and B2 phase, we first assume that the Poisson ratio, elastic anisotropy parameter, and $\gamma$ are approximately independent of pressure in each phase^34,35^, resulting in $\omega_{D}$ being proportional to $V^{1/6} K_{T}^{1/2}$ (Ref.^36^), where $K_{T}$ is the isothermal bulk modulus. This allows the thermal conductivity at a constant temperature to be simplified as $\Lambda (P) = A^{\prime}V^{5/6} K_{T}^{3/2}$, where $A^{\prime}$ is a constant determined by fitting our thermal conductivity data at ambient and 35 GPa for B1 and B2 phase, respectively, using their volume and isothermal bulk modulus. With the EoS of NaCl in B1^26^ and B2^21^ phase, respectively, we further compare the predicted pressure dependence of thermal conductivity based on the LS equation (blue dashed curves in Fig. 1) with our experimental data. Over a much wider range of compression that was previously inaccessible (i.e., ~ 35% volume compression in the NaCl B1 phase and after a pressure-induced structural transition, ~ 20% compression in the polymorphic B2 phase), the predicted NaCl thermal conductivities by the LS equation are in excellent agreement with our data for both B1 and B2 phase.

**Figure 3:**
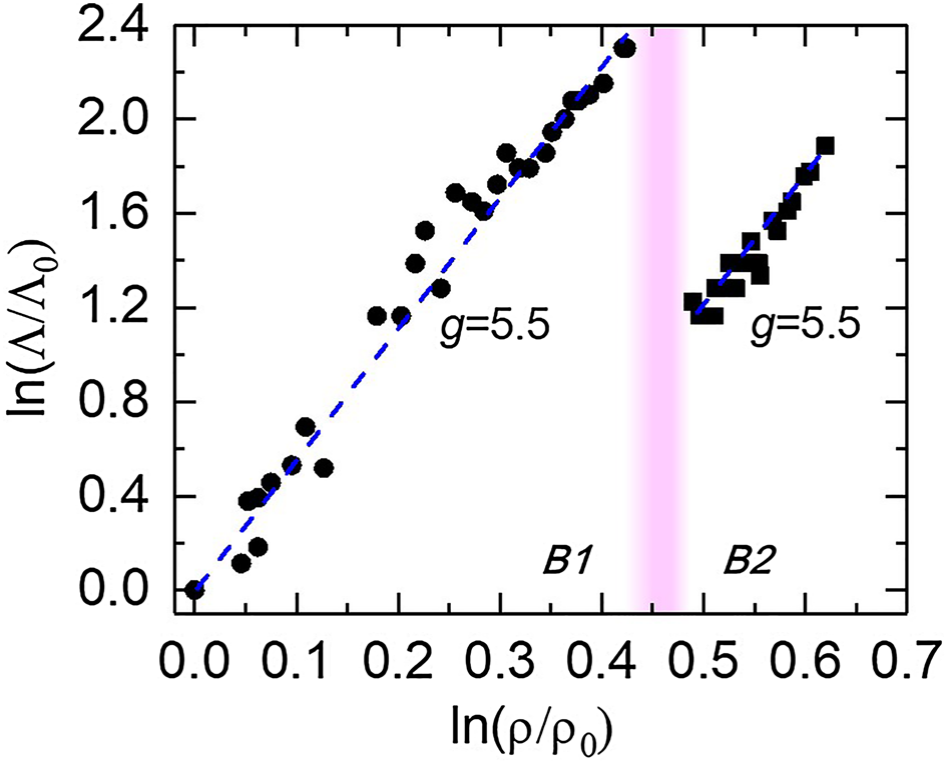
Logarithmic normalized thermal conductivity as a function of logarithmic normalized density. The blue dashed lines are linear fits to the data with a slope (*g*) of 5.5 for both B1 and B2 phase. The pink shaded region labels the B1–B2 transition.

Under the Debye approximation^16,37^, Eq. (1) can be equivalently expressed as a phenomenological description as a function of density ρ and temperature *T*:

2 $$ \Lambda \left( {\rho ,T} \right) = \Lambda_{0} \left( {\frac{\rho }{{\rho_{0} }}} \right)^{g} \left( {\frac{{T_{0} }}{T}} \right)^{n} , $$

where the $\Lambda_{0}$, $\rho_{0}$, and $T_{0}$ are the reference thermal conductivity, density, and temperature, respectively, and *n* here is close to −1 as we determined before (Fig. 2). The *g* = 3γ + 2q−1/3 (Ref. ^16,37^), where the Grüneisen parameter γ = (∂lnν/∂lnρ)_T_, q =  − (∂lnγ/∂lnρ)_T_, and ν is the phonon vibrational frequency. Figure 3 shows the ln(Λ/Λ_0_)-ln(ρ/ρ_0_) plot, where the $\Lambda_{0}$ and $\rho_{0}$ are the thermal conductivity and density at ambient conditions. The *g* was derived by linearly fitting the data: *g* = 5.5(± 0.2) and 5.5(± 0.5) for B1 and B2 phase, respectively. Our *g* values are in reasonable agreement with theoretical estimates^32,35^, while larger than those reported in Ref. ^32^ where the *g* value was determined by only two experimental data points in each phase. The similar *g* that we derived in B1 and B2 phase suggests that upon compression the behavior of phonon frequency change remains similar when transforming from the six- to eight-coordinated structure. Note that optical phonons and their scattering with acoustic phonons were proposed to contribute the thermal conductivity of NaCl^32,38^. The excellent agreement between our thermal conductivity data and prediction by the LS equation, however, suggests that, compared to the optical phonons and optical-acoustic phonon scattering, the acoustic phonons play major roles in heat transfer and the three phonon anharmonic scattering between acoustic modes controls the phonon mean-free-path in both B1 and B2 NaCl crystals.

**Figure 4:**
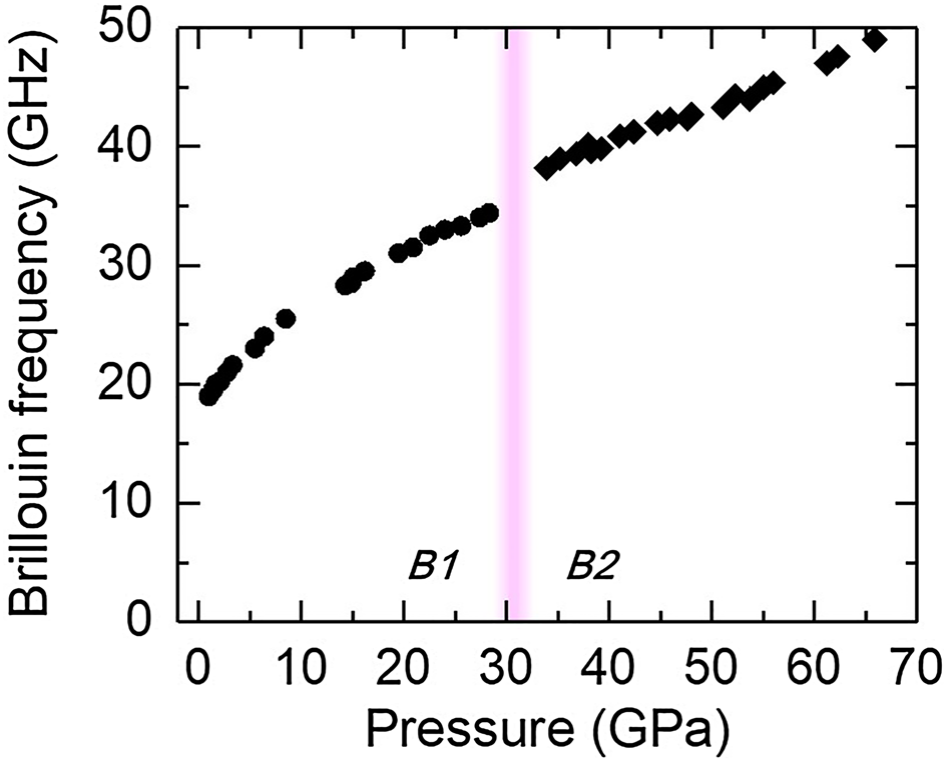
Pressure dependence of the Brillouin frequency of NaCl at room temperature. The frequency increases by about 10% across the B1–B2 transition (pink shaded region).

Figure 4 shows the Brillouin frequency *f* of NaCl as a function of pressure at room temperature. The *f* in B1 phase increases from 19 GHz at 1 GPa to 34.3 GHz at 28.2 GPa, and increases by ~ 10% across the B1–B2 transition around 30 GPa. To determine the compressional sound velocity *V*_p_ via *f* = 2*NV*_p_/λ in our backscattering geometry (*N* is the index of refraction and λ is the laser wavelength, see Methods for details), we first calculated the pressure dependence of density ρ from the EoS of NaCl in B1^26^ and B2^21^ phase, respectively. We then estimated the index *N* of NaCl from the Lorentz-Lorenz relation: (*N*^2^−1)/[ρ(*N*^2^ + 2)] = *B*, where *B* is assumed to be a constant. At ambient pressure, *N* = 1.53, ρ = 2.16 g cm^−3^, and *B* = 1.43 × 10^–4^; *N* = 1.83 at 20 GPa and 2.06 at 40 GPa.

**Figure 5:**
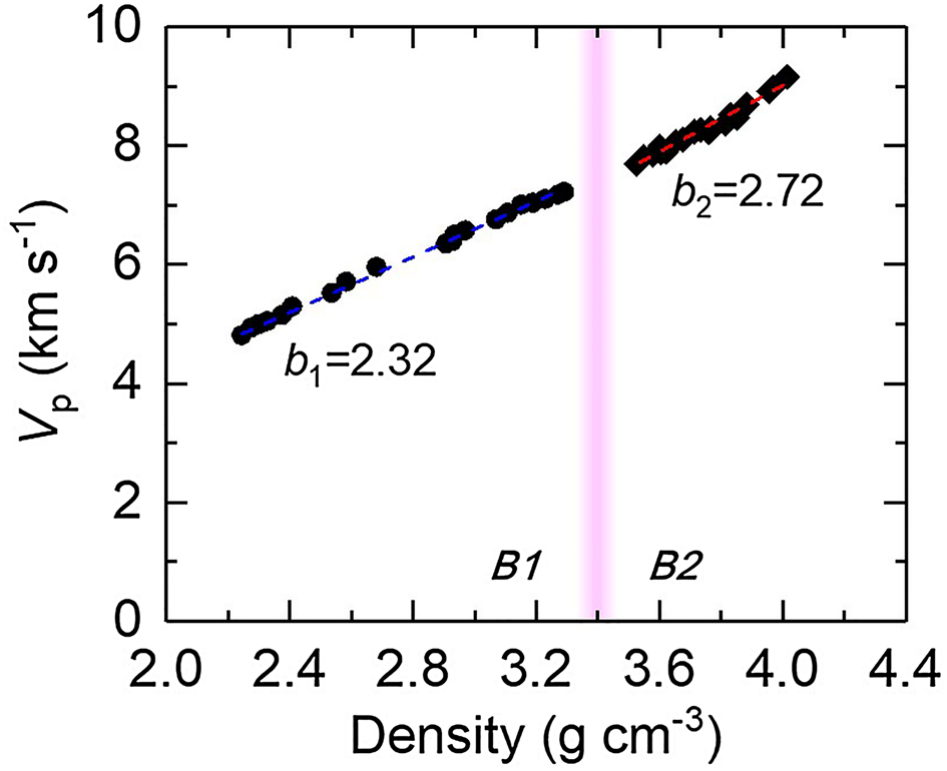
Compressional velocity *V*_p_ of NaCl as a function of density. The *V*_p_ is derived from the Brillouin frequency data (Fig. 4) and the refractive index *N* by the Lorentz-Lorenz equation (see the text for details). The density is calculated by the equations of state of NaCl in B1 and B2 phases. The *V*_p_ approximately linearly scales with the density, following the Birch’s law, with a slope of b_1_ = 2.32 (blue dashed line) and b_2_ = 2.72 (red dashed line) in B1 and B2 phase, respectively. The pink shaded region labels the B1–B2 transition.

We present the compressional velocity *V*_p_ as a function of density for B1 and B2 phase in Fig. 5. Both are consistent with literature results^25,26,28^. We further test the applicability of Birch’s law to NaCl over the density range we studied. The Birch’s law was originally formulated by Birch^39,40^, describing that the *V*_p_ of a mineral is linearly proportional to its density ρ in a form of *V*_p_ = *a*(m) + *b*ρ, where *a*(m) is a parameter associated with the atomic weight of the material and *b* is a constant. This relationship has been widely used to predict the profiles of sound velocity, density, and composition in Earth and planetary interiors^39,41^. It was later shown that within a certain range of density ρ, the Birch’s law may be treated as a linearization of a power law between the *V*_p_ and ρ^41–43^. Here we found that the *V*_p_ of NaCl B1 and B2 phase both follow the Birch’s law, as each of them scales approximately linearly with the density with a slope of 2.32(± 0.04) for B1 phase, in good agreement with previous results^26,28^, and 2.72(± 0.12) for B2 phase, respectively. Though a discontinuity in *V*_p_ across a pressure-induced structural transition has been observed in a number of materials, such as KCl^28^ and several minerals in Earth’s mantle^41^, our data show that across the B1–B2 transition, the *V*_p_ in NaCl B2 phase is only ~ 0.1 km s^−1^ (comparable to the measurement uncertainty) slower than that would be predicted by an extrapolation from the B1 phase following the Birch's law. We conclude that the Birch’s law is applicable to not only the B1 phase of NaCl, but also its B2 phase across a wide range of density we studied. Even though the interatomic potential and lattice dynamics that control the elasticity^42^ of NaCl are different in B1 and B2 phase, their density dependence of compressional velocity remain in the linear regime over the large volume compression.

## Conclusion

Using the combination of ultrafast time-domain thermoreflectance, picosecond interferometry, and DAC techniques, we have precisely determined the values of thermal conductivity and compressional velocity of NaCl, a common, important pressure medium for DAC experiments, to 66 GPa. With the EHDAC, we have also confirmed that the NaCl’s thermal conductivity follows the *T*^−1^ dependence at high pressures, typical of the pure crystal. Theses results enable us to conclude that the LS equation well describes the pressure evolution of the thermal conductivity of NaCl in both B1 and B2 phase, demonstrating that the LS equation is applicable to a pure crystal and its high-pressure polymorph over a much wider range of volume compression than previously accessible. In additional to providing insights to the physics of thermal transport and lattice dynamics in a simple crystal of NaCl, we also offer important database for the thermal conductivity of a critical pressure medium at high *P–T* conditions. Our results are significantly important and useful to accurately model the heat transport in a high *P–T* DAC experiment loaded with NaCl as the pressure medium, benefiting a variety of studies in materials physics and geosciences under extreme conditions.

## Methods

### Sample preparation

To perform high-pressure, room-temperature measurements, a thin sheet of borosilicate glass (D 263® T eco from Schott AG^44^) was first polished down to a thickness of ~ 10 μm, coated with ~ 90 nm thick Al film, and then loaded, along with several ruby spheres, into a symmetric piston-cylinder diamond-anvil cell (DAC) with a culet size of 300 μm and a Re gasket. The borosilicate glass was used as a reference substrate and compressed by loading NaCl powder (polycrystal, thermally dried at ~ 120℃ for one hour before being loaded) as the pressure medium and the sample of interest. A schematic illustration of the sample geometry and experimental setup is shown in Supplementary Information Fig. S1. The pressure within the DAC was determined by ruby fluorescence^45^, and the uncertainty of the pressure measurements at room temperature was typically < 5%. At pressures higher than about 50 GPa, the uncertainty was estimated by comparing the pressures derived from the Raman spectra of the ruby and diamond anvil^46^. The difference is typically < 3–5 GPa, depending on the pressure range. Example comparison of the pressure characterized by ruby fluorescence and Raman spectra of ruby and diamond at high pressures is shown in Supplementary Information Fig. S2.

To achieve simultaneous high pressure–temperature (*P–T)* conditions, we employed an externally (resistively) heated DAC (EHDAC)^47^, in which the diamond anvils were surrounded by a ring heater within a BX-90 DAC. The EHDAC provides an efficient means to reach stable and homogeneous high temperature conditions. Details of the assemblage of the EHDAC were described in Ref. ^47^. Using the EHDAC, we have performed thermal conductivity measurements up to 18.3 GPa and ~ 773 K. Given a temperature measurement uncertainty of about few Kelvin when using an R-type thermocouple, we estimate the resulting temperature-induced pressure uncertainty to be ~ 1% following the characterization method of ruby fluorescence described in Ref. ^48^. During the heating, the pressure variation was monitored by the ruby fluorescence where the temperature effect is calibrated following Ref. ^48^, see an example in Supplementary Information Fig. S3.

### Lattice thermal conductivity measurements

The thermal conductivity of NaCl at high pressure and room temperature was measured by time-domain thermoreflectance (TDTR) coupled with the DAC. Thermal conductivity measurements at simultaneous high *P–T* conditions were performed by TDTR coupled with the EHDAC. TDTR is a well-developed ultrafast optical pump-probe metrology which enables thermal conductivity measurements to pressures over 100 GPa with high precesion^7,49,50^. In our TDTR setup, we split the output of a Ti:sapphire oscillator into pump and probe beams. The pump beam heated up the Al film coated on the borosilicate glass, resulting in changes of temperature and optical reflectivity. The probe beam then detected the temporal evolution of the reflectivity change. We used a Si photodiode along with an RF lock-in amplifier to monitor the in-phase *V*_*in*_ and out-of-phase *V*_*out*_ components of the variation of the reflected probe beam intensity. The details of the TDTR were described in Ref.^51,52^.

We determined the thermal conductivity of NaCl by comparing the numerical calculations of a bi-directional thermal model with our data for the ratio –*V*_in_/*V*_out_ as a function of delay time between pump and probe beams. Our bi-directional thermal model simulates the temporal evolution of heat flow into the NaCl and the borosilicate glass within the DAC. Its detailed mathematical equations were described in Ref.^53^. Example data for TDTR measurements along with thermal model fitting are shown in Supplementary Information Fig. S4. There are several parameters in the thermal model, including laser spot size, and thickness, heat capacity, and thermal conductivity of each layer (i.e., NaCl, Al, and borosilicate glass), while the thermal conductivity of NaCl is the only significant unknown parameter that will be determined by fitting the TDTR data. The laser spot size (radius of the 1/e^2^ intensity) is ~ 7.6 μm. We used picosecond acoustics to determine the Al thickness at ambient conditions^54^; the acoustic echo signal, however, became too weak to be used to determine the Al thickness after the sample was compressed within the DAC. We thus estimated the changes in Al thickness at high *P–T* conditions using a method described in Ref.^3^ along with the thermal expansion of Al film (thermal expansion coefficient of 2.3 × 10^–5^ K^−1^) upon heating. Since the pump beam is electro-optically modulated at 8.7 MHz, the thermal penetration depths (a skin depth that a heat wave can propagate into a material) in the NaCl and borosilicate glass are both on the order of hundreds of nanometers^10^, making the thermal model calculations being insensitive to their thicknesses (~ 10 μm) under high *P–T* conditions.

Since the thermal conductivity of Al film at ambient conditions is high (~ 200 W m^−1^ K^−1^) and its variation at high *P–T* conditions has minimal effects on the thermal model calculations, we fixed the conductivity of Al as a constant under high *P–T* conditions. As described in Ref.^10^, we estimated the heat capacity of Al at high *P–T* conditions based on its atomic density, elastic constants, and Debye temperature. The thermal conductivity of borosilicate glass at high pressure and room temperature was separately measured using silicone oil^4^, instead of NaCl, as the pressure medium by the same experimental method used in the present study (Supplementary Information Text 1 and Fig. S5). Prior studies showed that when approaching the high temperature limit before being melted, the thermal conductivity of amorphous materials, including various glasses, is weakly dependent on the temperature^55–57^. Therefore, at a given pressure, the thermal conductivity of borosilicate glass was treated as a constant upon heating. Note that the glass transition temperature of borosilicate glass at ambient pressure is ~ 830 K^44^, higher than the maximum temperature (773 K) in our high *P–T* measurement, and is expected to increase as pressure increases. The volumetric heat capacity of borosilicate glass at ambient conditions is 2.06 J cm^−3^ K^−1^. Under high *P–T* conditions, it was estimated to be a constant following a method described in Ref.^10^: upon compression the heat capacity per molecule of borosilicate glass decreases, but its molecular density increases, counterbalancing these two effects. The high temperature here plays an opposite role in affecting these two effects, leading to a nearly constant volumetric heat capacity. Similarly, the volumetric heat capacity of NaCl at high *P–T* conditions is assumed to be approximately a constant of 1.84 J cm^−3^ K^−1^ over the *P–T* range we studied. Compared to the NaCl sample, the relatively low thermal conductivity of borosilicate glass (Fig. S5) substantially enhances the measurement accuracy. We estimated that the uncertainties in all the parameters used in our thermal model would propagate ~ 20% error in the derived thermal conductivity of NaCl before 30 GPa, and ~ 20–30% error at 30–66 GPa, see Supplementary Information Fig. S6, S7 and Ref.^58,59^ for details of the TDTR sensitivity analysis and uncertainty evaluations.

### Brillouin frequency measurements

We measured the Brillouin frequency of the NaCl at high pressure and room temperature using time-domain stimulated Brillouin scattering^10,60^, a picosecond interferometry based on the mechanism of inelastic light scattering from acoustic phonons. For longitudinal modes in our backscattering experimental geometry, the Brillouin frequency *f* = 2*NV*_p_/λ, where *N* is the index of refraction, *V*_p_ the compressional (longitudinal) sound velocity, and λ = 785 nm the laser wavelength. The typical uncertainty in the measurement of Brillouin frequency is ~ 1%. Example data for the Brillouin frequency measurements are shown in Supplementary Information Fig. S8.

### Data availability

The data that support the findings of this study are available from the corresponding author upon reasonable request.

## Supplementary Information

Supplementary Information.
