# Supplementary material for: High-pressure thermal conductivity and compressional velocity of NaCl in B1 and B2 phase
Source: Sci Rep. 2021 Oct 29;11:21321. doi: 10.1038/s41598-021-00736-2 (PMC8556477; doi:10.1038/s41598-021-00736-2)
Supplement: Supplementary file 1 — Supplementary Information. [file 41598_2021_736_MOESM1_ESM.pdf]

Supplementary Information for

High-pressure thermal conductivity and compressional velocity of NaCl in B1 and B2 phase

Wen-Pin Hsieh[1,2]

[1]*Institute of Earth Sciences, Academia Sinica, Nankang, Taipei 11529, Taiwan*

[2]*Department of Geosciences, National Taiwan University, Taipei 10617, Taiwan*

This Supplementary Information contains one supplementary text, eight supplementary figures, and two supplementary tables as shown below.

Supplementary Text 1.

Measurement of the thermal conductivity of borosilicate glass

The thermal conductivity of borosilicate glass at high pressure and room temperature (Supplementary Information Fig. S5) was separately measured using silicone oil[1], instead of NaCl, as the pressure medium by the same experimental method used in the present study (see the experimental geometry shown in Supplementary Information Fig. S1, where the NaCl is replaced by silicone oil and the ring-shaped resistive heater and thermocouple are not used for room temperature measurement). The method to analyze the borosilicate thermal conductivity is essentially the same as that to analyze the NaCl, which is described in *Methods* section in the main text. The thermal conductivity and volumetric heat capacity of silicone oil are from Supplementary Information Ref [1]. The uncertainties in all the

parameters used in the thermal model would propagate ~10% error in the derived thermal conductivity of borosilicate glass before 30 GPa, and ~20% error at 70 GPa.

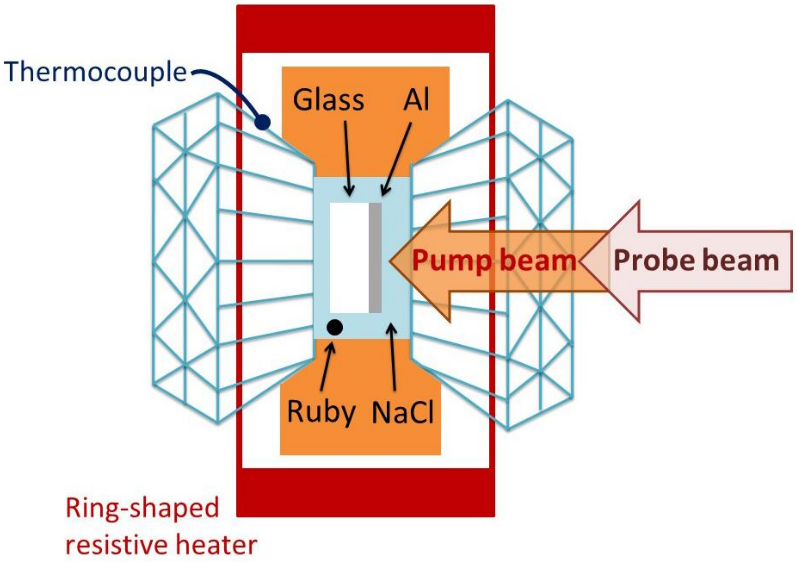

Supplementary Fig. S1. Schematic drawing of the optical pump-probe measurements (TDTR and picosecond interferometry) combined with a diamond-anvil cell. The borosilicate glass serves as a reference substrate, and NaCl is loaded as the pressure medium and sample of interest. An ~90 nm thick Al film is coated on the borosilicate glass and absorbs heat dumped by the pump beam. When performing high pressure-temperature measurements, a ring-shaped resistive heater is placed surrounding the sample chamber to heat up the sample. The temperature within the sample chamber is measured by an R-type thermocouple attached to a diamond anvil.

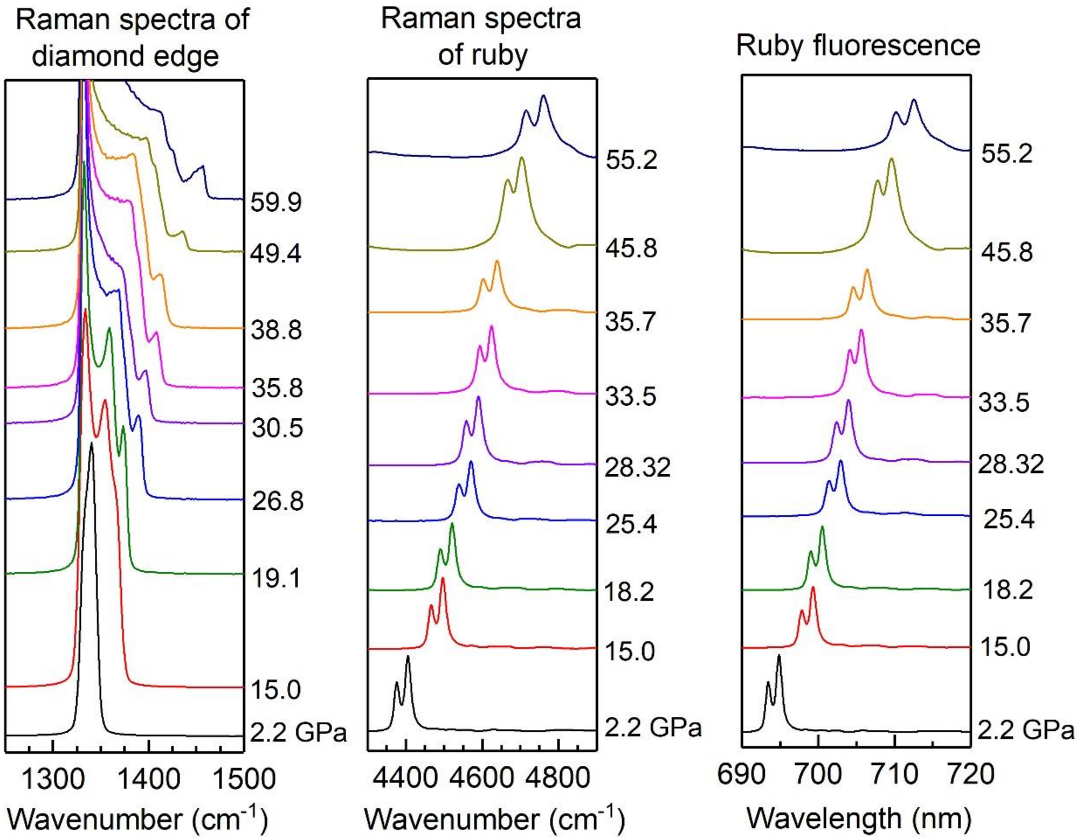

Supplementary Fig. S2. Example comparison of the pressure calibrated by Raman spectra of diamond edge (left panel), Raman spectra of ruby (center panel), and ruby fluorescence (right panel).

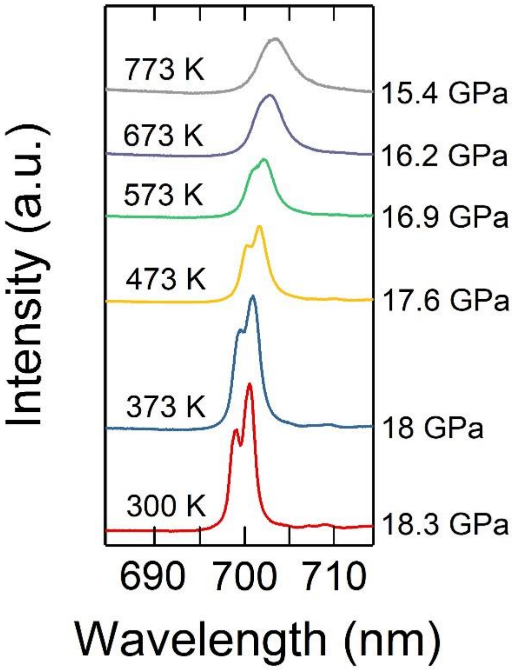

Supplementary Fig. S3. Example pressure variation upon heating monitored by the ruby fluorescence. The temperature effect is calibrated following Ref. [2].

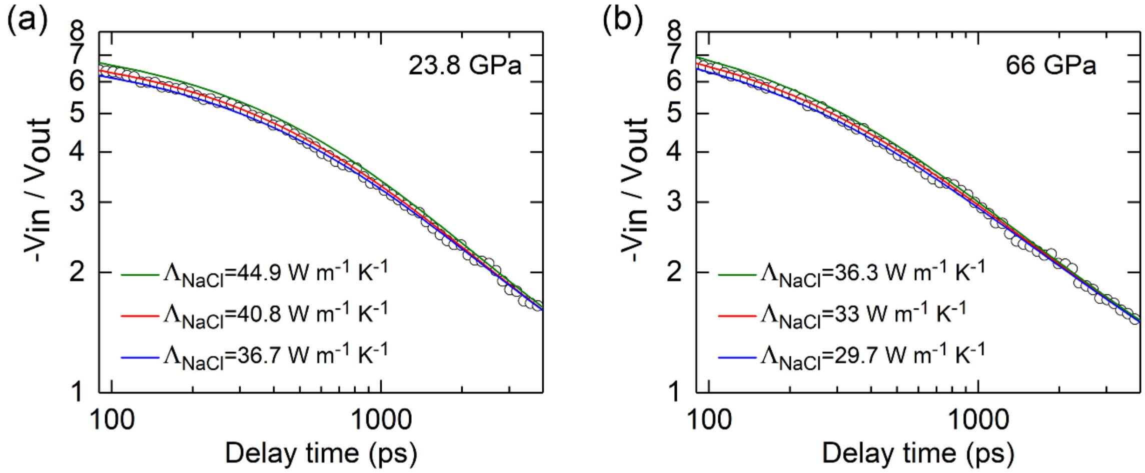

Supplementary Fig. S4. Comparison of representative TDTR data (open circles) with thermal model calculations (solid curves) for NaCl at (a**)** 23.8 and (b) 66 GPa. Different solid curves represent calculations using different input thermal conductivity of NaCl, Λ_{NaCl}. At 23.8 and 66 GPa, Λ_{NaCl}=40.8 and 33 W m^{-1} K^{-1} (red curves), respectively, give a best-fit to the data (see Supplementary Table S1 and S2 for other input parameters at 23.8 and 66 GPa, respectively). The ratio $-V_{in}$ /V_{out} is most sensitive to the Λ_{NaCl} during delay times of few hundred ps, particularly from ~200 to 500 ps[3,4]. A 10% change in Λ (green and blue curves) causes a clear deviation from the best-fit to the data, indicating our thermal model fitting and the derived NaCl thermal conductivity are precise and reliable due to the high quality data and sample geometry.

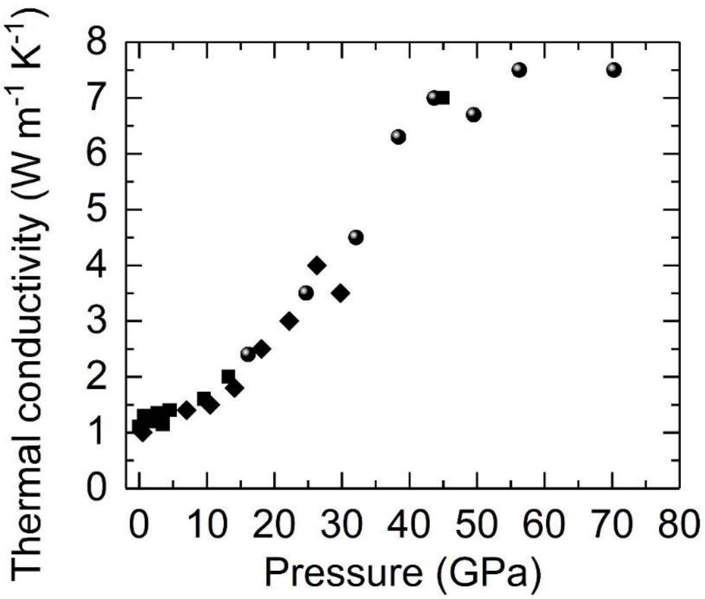

Supplementary Fig. S5. Pressure dependence of the thermal conductivity of borosilicate glass at room temperature. It was separately measured using silicone oil as the pressure medium by the same experimental method and geometry shown in Supplementary Fig. S1 without the ring-shaped resistive heater and thermocouple. The thermal conductivity of borosilicate glass remains relatively low and saturates to ~7.5 W m^{-1} K^{-1} after ~50 GPa. Different symbols represent different runs of measurement that yield consistent results.

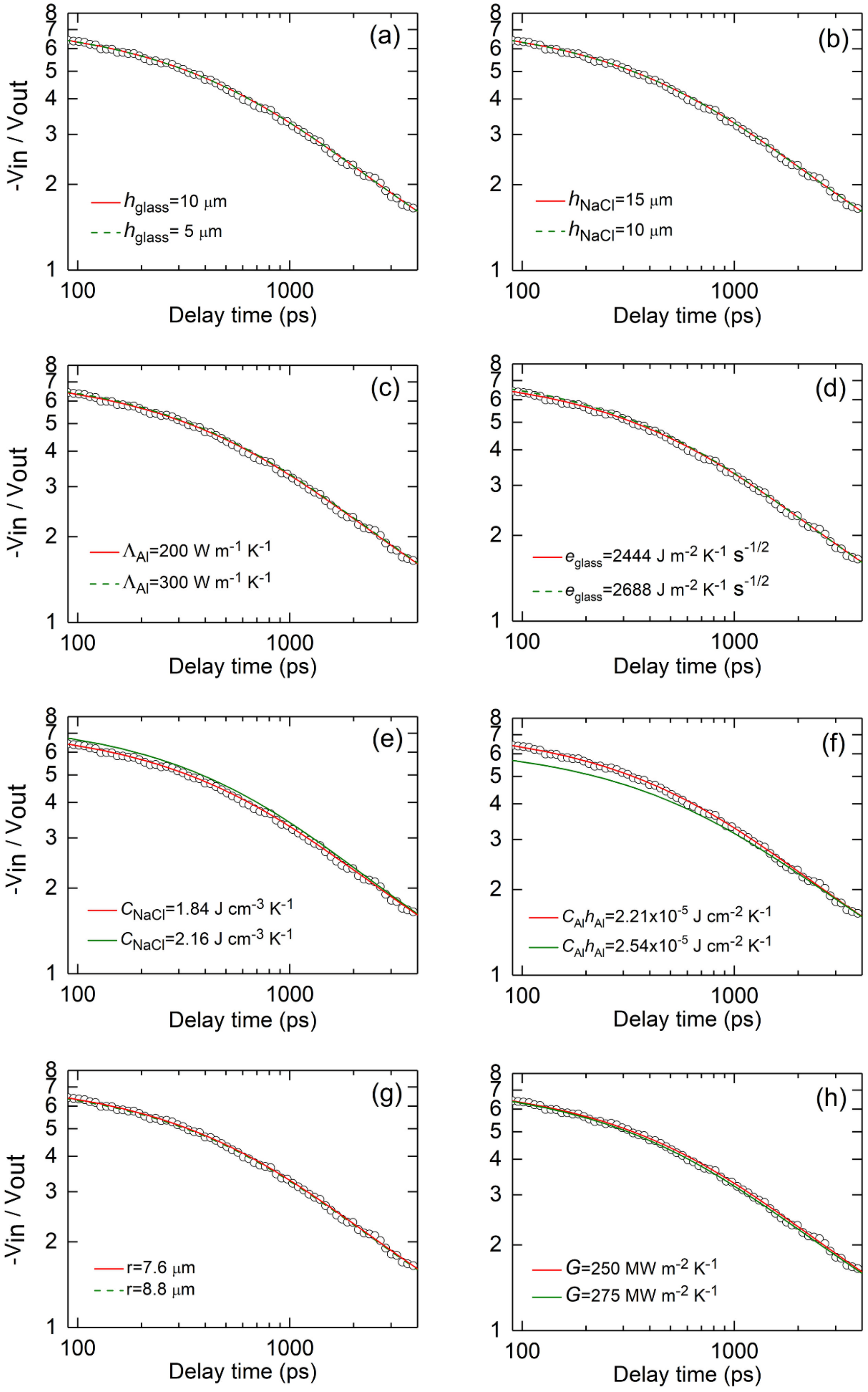
(a)
-Vin / Vout
Delay time (ps)
h_{glass}=10 μm
h_{glass}= 5 μm
(b)
h_{NaCl}=15 μm
h_{NaCl}=10 μm
(c)
Λ_{Al}=200 W m^{-1} K^{-1}
Λ_{Al}=300 W m^{-1} K^{-1}
(d)
e_{glass}=2444 J m^{-2} K^{-1} s$^{-1/2}$
e_{glass}=2688 J m^{-2} K^{-1} s$^{-1/2}$
(e)
C_{NaCl}=1.84 J cm^{-3} K^{-1}
C_{NaCl}=2.16 J cm^{-3} K^{-1}
(f)
$C_{Al}h_{Al}$=2.21x10^{-5} J cm^{-2} K^{-1}
$C_{Al}h_{Al}$=2.54x10^{-5} J cm^{-2} K^{-1}
(g)
r=7.6 μm
r=8.8 μm
(h)
G=250 MW m^{-2} K^{-1}
G=275 MW m^{-2} K^{-1}

Supplementary Fig. S6. Tests of sensitivity of the thermal model to input parameters for NaCl at 23.8 GPa in TDTR measurements. Here we fix the NaCl thermal conductivity Λ_{NaCl} to be 40.8 W m^{-1} K^{-1}, as derived in Supplementary Fig. S4(a), using input parameters listed in Supplementary Table S1. (a) and (b) The thermal model calculations show identical fits to the data even if the thicknesses of glass (h_{glass}) and NaCl (h_{NaCl}) change by as large as ~50%, respectively. This indicates that the uncertainties in the h_{glass} and h_{NaCl} have essentially no effect on the derived Λ_{NaCl}. (c) With a high Al thermal conductivity, Λ_{Al}, its uncertainty has a very minor effect on the Λ_{NaCl}. (d) An example variation in the thermal effusivity of the glass substrate, $e=(\Lambda_{glass}C_{glass})^{1/2}$, by 10% still gives nearly the same model calculation, indicating that its uncertainty does not influence the derived Λ_{NaCl}. (e) If the volumetric heat capacity of NaCl, C_{NaCl}, is uncertain by 17% (1.84 to 2.16 J cm^{-3} K^{-1}), the model calculation only slightly deviates from the data, which requires Λ_{NaCl} to decrease slightly to 37 W m^{-1} K^{-1} to re-fit the data, i.e., propagating approximately 10% uncertainty to the derived Λ_{NaCl}. (f) The major measurement uncertainty is from the uncertainty in Al heat capacity per unit area, product of volumetric heat capacity and thickness, $C_{Al}\,h_{Al}$, as the ratio $-V_{in}/V_{out}$ at few hundred ps delay time scales inversely with the $C_{Al}\,h_{Al}$ [3]. For instance, a 15% uncertainty requires approximately 20% change in the Λ_{NaCl} to re-fit the data. (g) Laser spot size changed by as large as 15% (7.6 to 8.8 μm) still shows the same model calculation, and thus does not affect the Λ_{NaCl}. (h) A 10% change in the thermal conductance of Al/NaCl interface and Al/glass interface, G, has a minor influence on the Λ_{NaCl}.

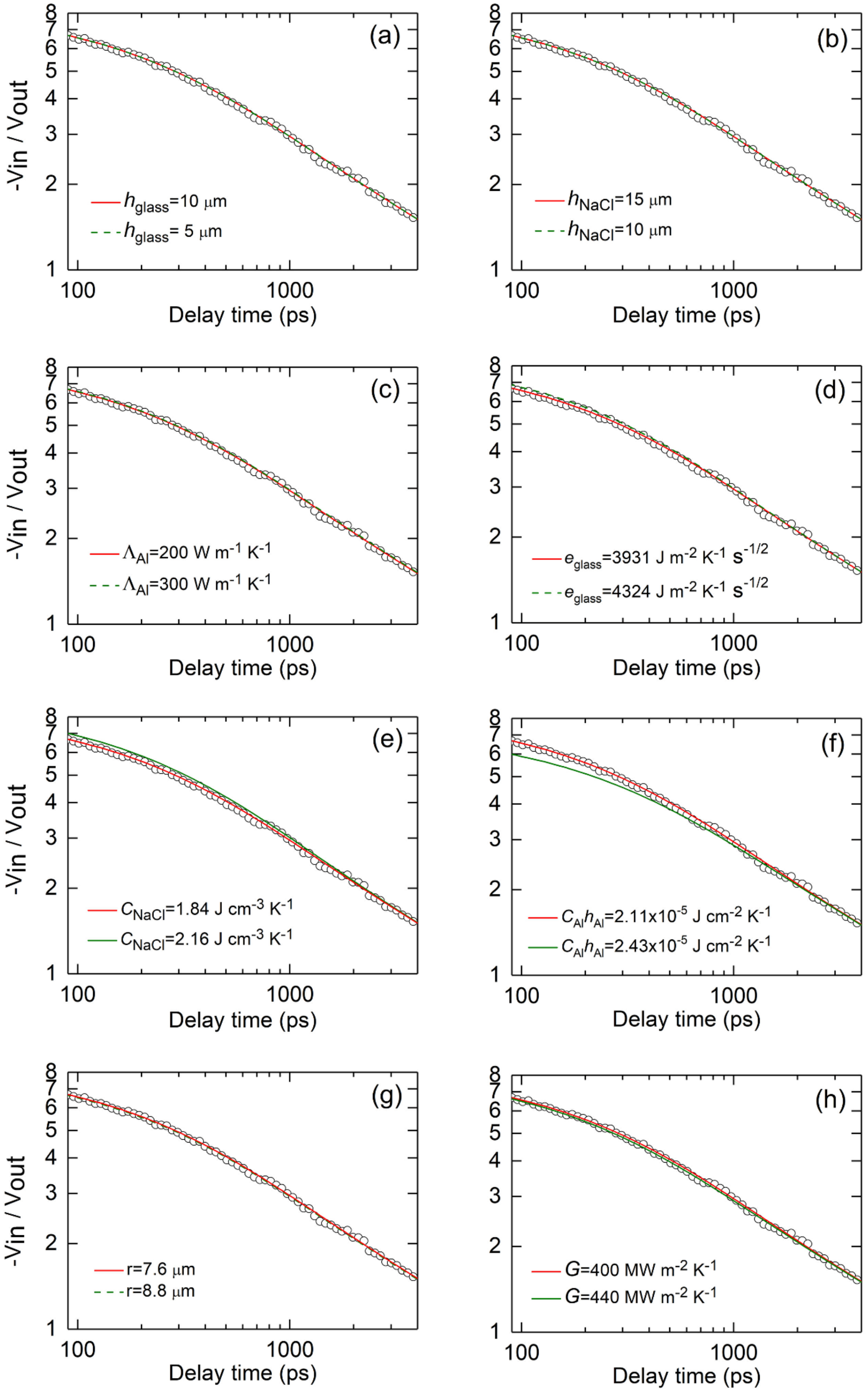
(a)
h_{glass}=10 μm
h_{glass}= 5 μm
(b)
h_{NaCl}=15 μm
h_{NaCl}=10 μm
(c)
Λ_{Al}=200 W m^{-1} K^{-1}
Λ_{Al}=300 W m^{-1} K^{-1}
(d)
e_{glass}=3931 J m^{-2} K^{-1} s$^{-1/2}$
e_{glass}=4324 J m^{-2} K^{-1} s$^{-1/2}$
(e)
C_{NaCl}=1.84 J cm^{-3} K^{-1}
C_{NaCl}=2.16 J cm^{-3} K^{-1}
(f)
$C_{Al}h_{Al}$=2.11x10^{-5} J cm^{-2} K^{-1}
$C_{Al}h_{Al}$=2.43x10^{-5} J cm^{-2} K^{-1}
(g)
r=7.6 μm
r=8.8 μm
(h)
G=400 MW m^{-2} K^{-1}
G=440 MW m^{-2} K^{-1}
-Vin / Vout
Delay time (ps)
1
2
3
4
5
6
7
8
100
1000

Supplementary Fig. S7. Tests of sensitivity of the thermal model to input parameters for NaCl at 66 GPa in TDTR measurements. Here we fix the NaCl thermal conductivity Λ_{NaCl} to be 33 W m^{-1} K^{-1}, as derived in Supplementary Fig. S4(b), using input parameters listed in Supplementary Table S2. (a) and (b) The thermal model calculations show identical fits to the data even if the thicknesses of glass (h_{glass}) and NaCl (h_{NaCl}) change by as large as ~50%, respectively, indicating that the h_{glass} and h_{NaCl} have very minor influences on the derived Λ_{NaCl}. (c) The high Al thermal conductivity has essentially no effect on the Λ_{NaCl}. (d) An example variation in the thermal effusivity of the glass substrate, $e=(\Lambda_{glass}C_{glass})^{1/2}$, by 10% still gives nearly the same model calculation. This indicates that its uncertainty does not affect the derived Λ_{NaCl}. (e) An example uncertainty of 17% (1.84 to 2.16 J cm^{-3} K^{-1}) for the NaCl volumetric heat capacity, C_{NaCl}, only slightly deviates the model calculation from the data, which requires Λ_{NaCl} to decrease slightly to 29 W m^{-1} K^{-1} to re-fit the data, i.e., propagating approximately 12% uncertainty to the derived Λ_{NaCl}. (f) The major measurement uncertainty is from the uncertainty in Al heat capacity per unit area, product of volumetric heat capacity and thickness, $C_{Al}\,h_{Al}$, as the ratio $-V_{in}/V_{out}$ at few hundred ps delay time scales inversely with the $C_{Al}\,h_{Al}$ [3]. For instance, if the $C_{Al}\,h_{Al}$ is uncertain by 15%, it requires approximately 25% change in the Λ_{NaCl} to re-fit the data. (g) Changes in laser spot size by as large as 15% (7.6 to 8.8 μm) do not affect the thermal model calculation and Λ_{NaCl}. (h) Thermal model calculation is not affected by a 10% change in the thermal conductance of Al/NaCl interface and Al/glass interface.

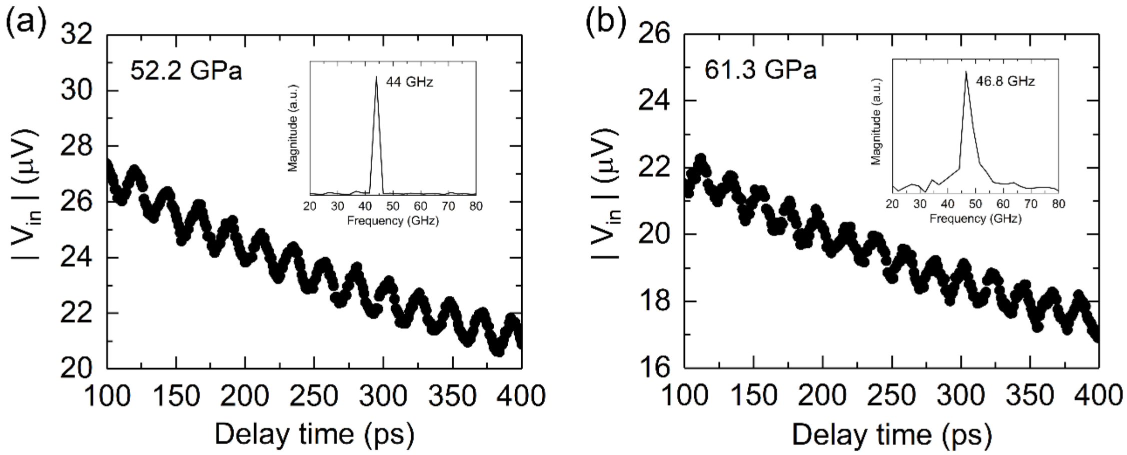

Supplementary Fig. S8. Example data for the picosecond interferometry on NaCl at (a) 52.2 GPa and (b) 61.3 GPa. We determined the Brillouin frequency by converting the periodic oscillations of V_{in} signal in time domain into frequency domain via Fourier transformation (insets). At 52.2 GPa, the Brillouin frequency is determined to be 44 GHz, and at 61.3 GPa, it is 46.8 GHz.

Supplementary Table S1. Input parameters in the thermal model for NaCl at 23.8 GPa and 300 K in TDTR measurements

P (GPa)	C_{NaCl} (J cm^{-3} K^{-1})	C_{Al} (J cm^{-3} K^{-1})	h_{Al} (nm)*	$e=(\Lambda_{glass}C_{glass})^{1/2}$ (J m^{-2} K^{-1} s$^{-1/2}$)	r (μm)	$h_{NaCl/glass}$ (μm)	Λ_{Al} (W m^{-1} K^{-1})	G (MW m^{-2} K^{-1})
23.8	1.84	2.643	83.8	2444	7.6	15/10	200	250

*In this experimental run, the Al thickness at ambient pressure is 89 nm.
C_{NaCl}: NaCl heat capacity, C_{Al}: Al heat capacity, h_{Al}: Al thickness, e: glass thermal effusivity, r: laser spot size, h_{NaCl}: NaCl thickness, h_{glass}: glass thickness, Λ_{Al}: Al thermal conductivity, G: thermal conductance of Al/glass and Al/NaCl interfaces.

Supplementary Table S2. Input parameters in the thermal model for NaCl at 66 GPa and 300 K in TDTR measurements

P (GPa)	C_{NaCl} (J cm^{-3} K^{-1})	C_{Al} (J cm^{-3} K^{-1})	h_{Al} (nm)*	$e=(\Lambda_{glass}C_{glass})^{1/2}$ (J m^{-2} K^{-1} s$^{-1/2}$)	r (μm)	$h_{NaCl/glass}$ (μm)	Λ_{Al} (W m^{-1} K^{-1})	G (MW m^{-2} K^{-1})
66	1.84	2.684	78.8	3931	7.6	15/10	200	400

*In this experimental run, the Al thickness at ambient pressure is 89 nm.
C_{NaCl}: NaCl heat capacity, C_{Al}: Al heat capacity, h_{Al}: Al thickness, e: glass thermal effusivity, r: laser spot size, h_{NaCl}: NaCl thickness, h_{glass}: glass thickness, Λ_{Al}: Al thermal conductivity, G: thermal conductance of Al/glass and Al/NaCl interfaces.

References

1. Hsieh, W.-P. Thermal conductivity of methanol-ethanol mixture and silicone oil at high pressures. *J. Appl. Phys.* **117**, 235901 (2015).
2. Datchi, F. *et al.* Optical pressure sensors for high-pressure-high-temperature studies in a diamond anvil cell. *High Press. Res.* **27**, 447–463 (2007).
3. Zheng, X., Cahill, D. G., Krasnochtchekov, P., Averback, R. S. & Zhao, J. C. High-throughput thermal conductivity measurements of nickel solid solutions and the applicability of the Wiedemann-Franz law. *Acta Mater.* **55**, 5177–5185 (2007).
4. Cahill, D. G. & Watanabe, F. Thermal conductivity of isotopically pure and Ge-doped Si epitaxial layers from 300 to 550 K. *Phys. Rev. B* **70**, 235322 (2004).
